# Mutations of the EPHB6 Receptor Tyrosine Kinase Induce a Pro-Metastatic Phenotype in Non-Small Cell Lung Cancer

**DOI:** 10.1371/journal.pone.0044591

**Published:** 2012-12-04

**Authors:** Etmar Bulk, Jun Yu, Antje Hascher, Steffen Koschmieder, Rainer Wiewrodt, Utz Krug, Bernd Timmermann, Alessandro Marra, Ludger Hillejan, Karsten Wiebe, Wolfgang E. Berdel, Albrecht Schwab, Carsten Müller-Tidow

**Affiliations:** 1 Department of Medicine A – Hematology, Oncology, Pneumology, University of Muenster, Muenster, Germany; 2 Institute of Physiology II, University of Muenster, Muenster, Germany; 3 Center of Teaching Experiment, School of Basic Medical Science, Fourth Military Medical University, Xi’an, Shaanxi, People’s Republic of China; 4 Max-Planck-Institute for Molecular Genetics, Berlin, Germany; 5 Department of Thoracic Surgery, Niels-Stensen-Hospital Ostercappeln, Ostercappeln, Germany; 6 Department of Thoracic Surgery, University of Muenster, Muenster, Germany; Queen Elizabeth Hospital, Hong Kong

## Abstract

Alterations of Eph receptor tyrosine kinases are frequent events in human cancers. Genetic variations of EPHB6 have been described but the functional outcome of these alterations is unknown. The current study was conducted to screen for the occurrence and to identify functional consequences of EPHB6 mutations in non-small cell lung cancer. Here, we sequenced the entire coding region of EPHB6 in 80 non-small cell lung cancer patients and 3 tumor cell lines. Three potentially relevant mutations were identified in primary patient samples of NSCLC patients (3.8%). Two point mutations led to instable proteins. An in frame deletion mutation (del915-917) showed enhanced migration and accelerated wound healing *in vitro*. Furthermore, the del915-917 mutation increased the metastatic capability of NSCLC cells in an *in vivo* mouse model. Our results suggest that EPHB6 mutations promote metastasis in a subset of patients with non-small cell lung cancer.

## Introduction

Lung cancer is a leading cause of cancer related death with the majority of cases belonging to the group of non-small cell lung cancer (NSCLC) [1], [2]. Development of distant metastasis is the major cause of NSCLC related death. Receptor tyrosine kinases (RTKs) play important roles in the metastatic process [3], [4]. One of the best known RTK associated with a metastasis phenotype, is the epidermal growth factor receptor (EGFR) with its family members ERBB2/Her2, ERBB3 and ERBB4. RTKs such as the EGFR family are therefore attractive targets for improved molecular therapy approaches in cancers [3], [5]. To date, the Ephrin (EPH) receptor subfamily is the largest subfamily of RTKs comprising of 16 members in vertebrates, namely EPHA receptors 1–10 (EPHA1-A10) and EPHB receptors 1–6 (EPHB1-B6) [6], [7]. EPHB receptors interact with the Ephrin family of ligands. Upon interaction with their Ephrin ligands, EPH receptors modulate a variety of biological activities, including cell-cell interaction and cell migration [8], [9]. Loss of the kinase-dead EPHB6 is associated with advanced tumor stages and cancer progression [10]–[16]. Several publications report on high EPHB6 expression being a favorable prognostic marker in neuroblastoma [10]–[12]. In addition, mRNA expression of EPHB6 was decreased in metastatic melanoma and in invasive breast cancer cell lines with metastatic potential [14]–[16]. Functionally, EPHB6 suppresses invasiveness, growth rate and colony-forming efficiency of cultured breast cancer cells [17]–[18], regulates cell adhesion and affects migration [19].

Previously, we identified several human RTKs whose expression level correlated with the development of metastasis in early-stage NSCLC [20]. Whereas high mRNA expression of several RTKs was associated with an increased frequency of metastasis development, high mRNA expression levels of the two RTKs EPHB6 and DKFZ1 indicated a reduced risk for metastasis [20]. Recently, we identified EPHB6 as an epigenetically silenced metastasis suppressor in NSCLC, and expression of EPHB6 prevented metastasis formation in a xenograft metastasis model [21].

Here, we scrutinized the EPHB6 variation by DNA sequencing, and characterized the functional consequences of EPHB6 mutations *in vivo* and *in vitro* with regard to their potential role in NSCLC metastasis.

**Figure 1:**
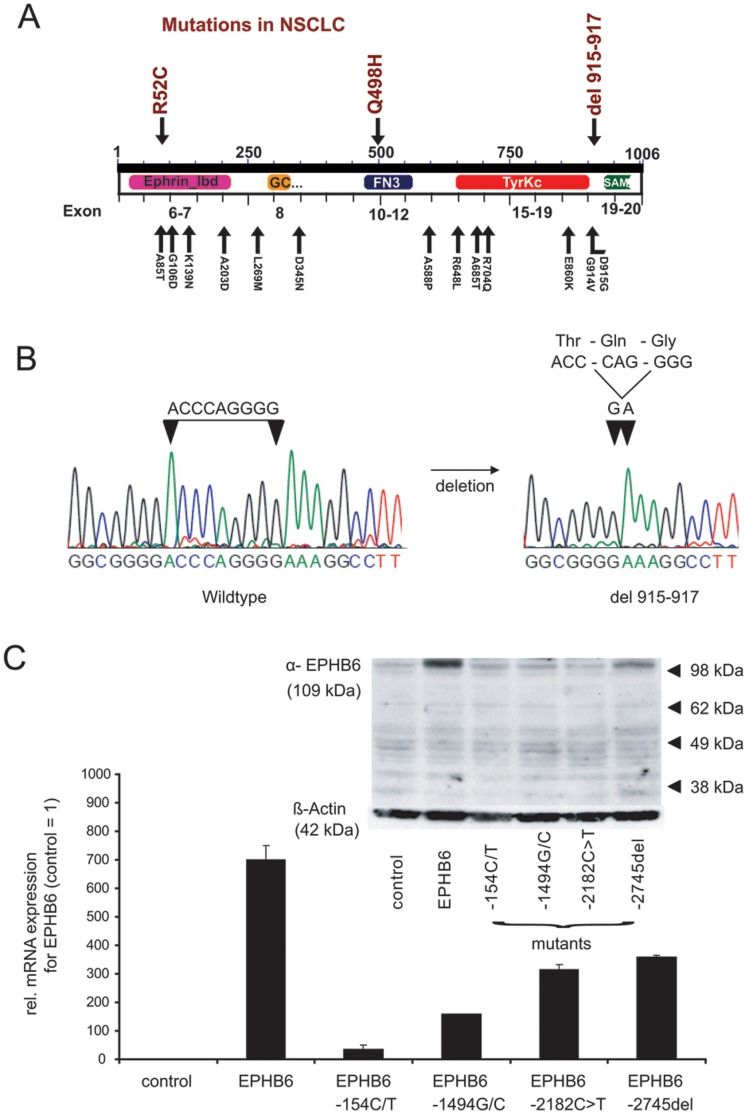
EPHB6 mutants and functional domains. A) Functional domains of the EPHB6 gene are shown in relation to their exons and to identified mutations for EPHB6. The description of the mutations correspond to their localization on the protein sequence. The mutations R52C, Q498H, and DPG915-917del were identified in NSCLC patient samples in current study. B) Electropherogram of the EPHB6-wildtype sequence and the deletion mutant for EPHB6. C) Expression levels of EPHB6-mutants in transfected cells. Bulk transfected cells were GFP sorted and expanded in selection media. Expression levels are shown for bulk cultures with >90% GFP expression. Differences of expression analysis between protein levels and mRNA levels are shown. For quantitative real-time PCR the average and standard deviation of three independent experiments are shown. The western blot shows a representative example.

## Materials and Methods

### Cell culture

The NSCLC cell lines involved in current study have been described previously [21]. Briefly, A549 lung adenocarcinoma cells were cultured at 37°C, high humidity and 5% CO_2_ in DMEM (Dulbeccós Modified Eagle’s medium, Invitrogen, Carlsbad, CA). The medium was supplemented with 10% fetal calf serum (FCS) and 1% streptomycin and penicillin. HTB56 and HTB58 lung adenocarcinoma cells were cultured at 37°C, high humidity, and 5% CO_2_ in MEM (Modified Eagle’s medium, Invitrogen, Carlsbad. CA). The medium was supplemented with 10% FCS, 1% streptomycin and penicillin, 1% glutamine, 1% sodium pyruvate, and 1% nonessential amino acid.Cell line identity was confirmed by STR-genotyping.

**Table 1:** Summary of non-synonymous mutations for EPHB6 (NM_004445 and NP_004436) found in tumors.

AA Mutation	CDS Mutation	Pubmed Id	Primary Tissue	Histology	Domains	Functional prediction	
						SIFT	PolyPhen
p.A685T	c.2053G>A	18772890	central nervous system	glioma	TyrKc	0.00	probably damaging with a score of **1.000**
p.E860K	c.2578G>A	16618716	central nervous system	glioma	TyrKc	0.04	possibly damaging with a score of **0.671**
p.G106D	c.317G>A	18772890	central nervous system	glioma	Ephrin_lbd	0.42	probably damaging with a score of **0.971**
p.A588P	c.1762G>C	16959974	large intestine	carcinoma	Between FN3 and TyrKc domains	0.22	probably damaging with a score of **0.983**
p.D345N	c.1033G>A	16959974	large intestine	carcinoma	Between GCC2_GCC3 and FN3 domains	0.00	probably damaging with a score of **0.999**
p.D915G	c.2744A>G	16959974	large intestine	carcinoma	Between TyrKc and SAM domains	0.42	benign with a score of **0.000**
p.G914V	2741 G>T	21351276	large intestine	carcinoma	Between TyrKc and SAM domains	0.22	probably damaging with a score of **0.998**
p.R704Q	c.2111G>A	16959974	large intestine	carcinoma	TyrKc	0.01	benign with a score of **0.004**
p.915_917del	c.2745del(CCCAGGGGA)	Not reported	lung	carcinoma	Between and Tyrkc and SAMdomains	not known	not known
p.A203D	c.608C>A	18948947	lung	carcinoma	Ephrin_lbd	0.01	probably damaging with a score of **0.999**
p.A85T	c.253G>A	18948947	lung	carcinoma	Ephrin_lbd	0.06	probably damaging with a score of **0.999**
p.K139N	c.417G>T	18948947	lung	carcinoma	Ephrin_lbd	0.27	probably damaging with a score of **0.999**
p.L269M	c.805C>A	18948947	lung	carcinoma	Between Ephrin_lbd and GCC2_GCC3 domains	0.25	possibly damaging with a score of **0.279**
p.Q498H	c.1494G/C	Not reported	lung	carcinoma	FN3	0.55	probably damaging with a score of **0.989**
p.R52C	c.154C/T	Not reported	lung	carcinoma	Ephrin binding	0.03	probably damaging with a score of **1.000**
p.P728H	c.2183C>A	20668451	lung	carcinoma	TyrKc	0.00	probably damaging with a score of **1.000**
p.R648L	c.1943G>T	18948947	lung	carcinoma	Between FN3 and TyrKc domains	0.32	probably damaging with a score of **0.993**
p.P728S	c.2182C>T	17344846	ovary	carcinoma	TyrKc	0.00	probably damaging with a score of **1.000**
p.L21F	c.63G>T	21720365	ovary	carcinoma	Ephrin_lbd	0.01	probably damaging with a score of **0.996**
p.R704W	c.2110C>T	19718025	skin	melanoma	TyrKc	0.00	probably damaging with a score of **0.989**
p.G404S	c.1210G>A	19718025	skin	melanoma	FN3	1.00	benign with a score of **0.000**
p.A688G	c.2063C>G	19718025	skin	melanoma	TyrKc	0.00	possibly damaging with a score of **0.662**
p.S152F	c.455C>T	19718025	skin	melanoma	Ephrin_lbd	0.05	possibly damaging with a score of **0.881**
p.R679Q	c.2036G>A	19718025	skin	melanoma	TyrKc	0.44	probably damaging with a score of **0.993**
p.S131F	c.392C>T	21499247	skin	melanoma	Ephrin_lbd	0.76	benign with a score of **0.034**
p.Q756R	c.2267A>G	21097718	stomach	carcinoma	TyrKc	0.06	probably damaging with a score of **0.998**

Note: The table contains data from the databases of http://www.sanger.ac.uk/genetics/CGP/cosmic/, http://strubiol.icr.ac.uk/extra/mokca, and the references were listed in the column of “Pubmed Id”. The NSCLC mutations identified in this study were marked as “not reported”. Two sequence homology-based tools were used to predict the potential impact of the identified non-synonymous substitutions on protein function: Sort Intolerant from Tolerant (SIFT; http://sift.bii.a-star.edu.sg/) and Polymorphism Phenotype (PolyPhen-2; http://genetics.bwh.harvard.edu/pph2/). If the SIFT prediction tolerance index score was less than 0.05, the variation was considered possibly damaging. Predictions made by PolyPhen-2 were assigned as “probably damaging,” “possibly damaging” or “benign.” Deletion mutations cannot be tested by either SIFT or PolyPhen-2.

### Patient Specimens

Primary tumor specimens and tumor-free lung tissues were obtained at the time of initial surgery from 80 patients with histology-proven NSCLC at a University hospital in Germany. Samples were immediately shock frozen and stored in liquid nitrogen. The tumor samples were checked for the percentage of tumor cells by histology, and only tumor biopsies with at least 70% cancer cells were used for subsequent analyses. Similarly, cancer-free control samples were also confirmed by histological examination. All patients provided written consent and the study was approved by the Ethics committee at the University of Münster.

**Figure 2:**
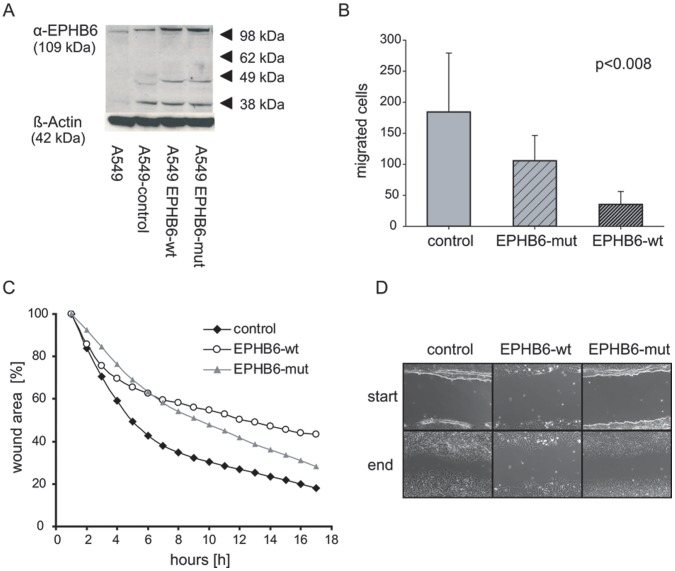
Migration analysis of EPHB6 expressing NSCLC cells. A) Protein expression of stably transfected A549 cell lines expressing wild type EPHB6 or the EPHB6 deletion mutant. Cells were co-transfected using an EGFP -pcDNA3.1^+^ vector for identification of selected clones. Multiple clones were pooled and further selected as bulk cultures. B) Transwell migration assays were performed with empty vector control cells, EPHB6 mutant and EPHB6 wildtype cells. Five different experiments in triplicates were analyzed. *: significant (p<0.05) differences by (EITHER ANOVA OR t-test) The provided p-value between the three different cell lines was statistically analyzed from all migrated cells by using the OneWay ANOVA-test. The analysis of the pair-wise t-test results in a significant p-value for the control cells vs. EPHB6-wt cells (p<0.015) and between the EPHB6-wt cells and the EPHB6-mut cells (p<0.005). C) *In vitro* wound healing scratch assay. Cells were scratched by a 10 µl pipette tip. The scratch areas were recorded over a periode of 17 hours. Shown are means of three different experiments, calculated as percentage from one initial point for all three cell lines. The ANOVA-test (p<0.002) indicated statistically significant differences between the three cell lines. D) Representative images of the scratch assays at the beginning and the end of the experiments.

**Figure 3:**
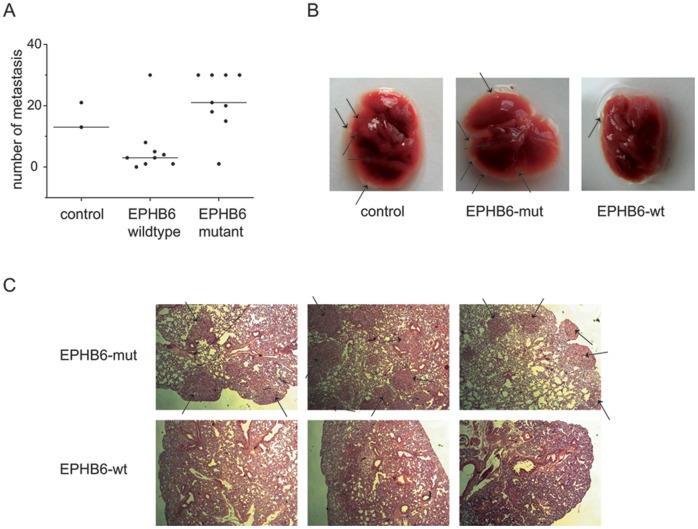
Development of metastasis *in vivo*. A) Number of pulmonary metastases in evaluable NOD/SCID mice four weeks after transplantation, each with 3×10^5^ stably transfected A549 cells expressing EPHB6-wt (n = 9), EPHB6-del915-917 (n = 9) or empty vector control cells (n = 2). Dots represent individual mice and horizontal lines the median value of metastases. B) Images from representative whole lungs of NOD/SCID mice, transplanted with A549 cells expressing EPHB6-wt, EPHB6-del915-917, or empty vector control. Lung metastases are marked by black arrows. C) Images from lung sections of NOD/SCID mice, stained with hematoxylin. Metastases are marked by black arrows. Three representative examples are shown each for mice transplanted with A549 cells expressing EPHB6-wt or EPHB6-del915-917.

**Figure 4:**
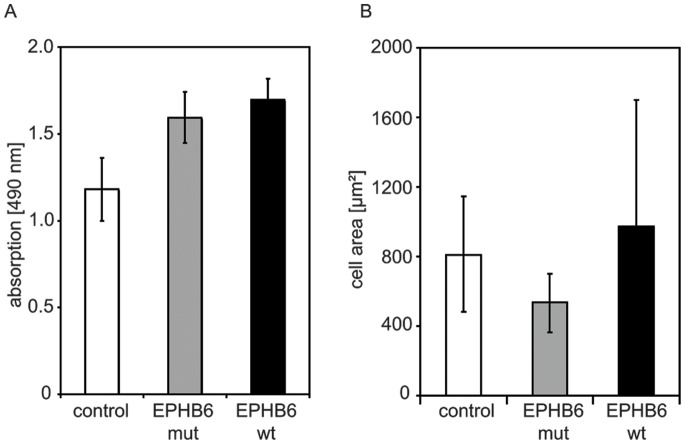
Proliferative activity and cell size of EPHB6 wildtype and mutant cells. A) Proliferative activity of empty vector control, EPHB6 wildtype and mutant cells were analyzed using a colorimetric MTT assay after 72 hours. Data are shown as means +/− standard deviation of three independent experiments. Differences were statistically not significant (ANOVA). B) Cell size of individual cells (n = 20) growing on plastic dishes was analyzed by live video microscopy and recorded. EPHB6 mutant cells showed a significantly reduced cell size in comparison to EPHB6 wild type and to control cells (p<0.05, t-test).

### EPHB6 Sequencing

Genomic DNA was extracted using DNAzol (Invitrogen, Carlsbad, CA, USA). Primers were designed with Primer3 software (DISTRIBUTOR) to amplify polymerase-chain-reaction (PCR) fragments sized between 400 and 800 bps and covering the complete coding region of the EPHB6 gene (details of PCR are provided in Supplementary Material). All All fragments were amplified by PCR with Taq DNA Polymerase (total reaction volume 20 µl) supplemented with a home-made PCR enhancer as described [22]. Both strands were sequenced utilizing the PCR primers. Additional internal primers were used for PCR products longer than 600 bp to ensure double-stranded sequence information for the whole PCR fragment. Sequencing was performed on ABI3730xl automated DNA sequencers with the BigDye Terminator V3.1 Cycle Sequencing Kit (Applied Biosystems). The sequenced coding region of *EPHB6* was compared with the reference sequence (GenBank accession No. NM_004445).

### Site-directed Mutagenesis

The coding region of the human EPHB6 cDNA (base 833-3853 NCBI Accession No. NM_004445) was cloned into the pcDNA4 To/myc/hisA expression vector (Invitrogen, Carlsbad, CA, USA). Mutations in the coding sequence of EPHB6 were introduced with the QuickChange XL site-directed mutagenesis kit (Stratagene, La Jolla, CA, USA) using primers with the sequences: forward (5′- AGGCTGGCGGGGAAAGGCCTTCCCAGG), reverse (5′- CCTGGGAAGGCCTTTCCCCGCCAGCCT) and using pcDNA4-EPHB6 vector as the template. Afterwards, the correct sequence was verified by sequencing. Primers for further mutations will be provided upon request.

### Expression Constructs and Transfection

Human A549 cells were co-transfected using the transfection reagent Nanofectin (PAA, Austria) according to the manufacturer’s protocol. Co-transfection was carried out with either pcDNA4 (empty vector control), wild type EPHB6 expression construct (pcDNA4-EPHB6-wt) or EPHB6 mutant expression constructs, each with an EGFP expressing vector construct (pcDNA3.1-GFP, expressing enhanced green fluorescent protein EGFP) for selection and identification of transfected cells. Transfected cells were selected with 700 µg/ml G418 (Sigma, St. Louis, MO, USA) and 400 µg/ml Zeocin (Invitrogen, Carlsbad, CA, USA). Bulk cultures were FACS sorted for GFP-expression and expanded. In addition to bulk cultures, we also pooled multiple high GFP expressing clones to obtain sufficient expression levels. Expression was verified by Western blotting and real-time RT-PCR.

### RNA Isolation and Reverse Transcription

Total RNA was isolated using TRIzol reagent (Invitrogen, Carlsbad, CA, USA). A total amount of 1 µg of RNA from each sample was reverse-transcribed using random primers and MMLV reverse transcriptase according to the manufacturer’s protocol (Promega, Madison, Wisconsin, USA).

### Gene Expression Analyses by Quantitative Real-time RT-PCR

For quantitative real-time RT-PCR, cDNA was amplified in an ABI Prism 7700 sequence detector (Applied Biosystems, Foster City, CA, USA). EPHB6 was detected with the following primers and probe: forward (5′-TGGACTATCAGCTCCGCTACTATG), reverse (5′- GTGGCAGTGTTGGTCTCGC) and probe (5′-FAM- CCAGGCAGAAGACGAATCCCACTCCTT-TAMRA). The relative amounts of gene expression were calculated by using the expression of GAPDH as an internal standard.

### Western Blot Analysis

Proteins were detected using the following antibodies: anti-human EPHB6 (1 µg/ml, Abnova Corporation, Neihu, Taipei, Taiwan, or ABGENT, San Diego, CA, USA) and β-actin (40 ng/ml, Sigma, USA) as primary antibodies, Goat anti-mouse and Goat anti-rabbit (both from Dianova, Hamburg, Germany) as secondary antibodies. Western blot analysis was carried out as described [23]. The *See Blue Plus2* protein marker (Invitrogen) was used as a size indicator.

### Boyden Chamber Assay

A total of 5×10^5^ A549 cells (in 100 µl DMEM with 5% FCS) were seeded into the upper part of a Transwell® chamber (transwell filter inserts in 6.5 mm diameter with a pore size of 5 µm, Corning Inc., Corning, NY), which was 30 min precoated with 50 µg fibronectin. In the lower part of the chamber 600 µl DMEM medium with 20% FCS (a serum gradient was used as chemoattractant) was added and the assay was performed for 16 hours at 37°C and 5% CO_2_ before migrated cells were analyzed by flow cytometry. All assays were repeated four times and independently performed in triplicates.

### In vitro Wound Healing – Scratch Assay

A549 cells were seeded in a 25-mL tissue culture flask at a density of 350,000 cells per flask and were cultured over a period of three days. Confluent cell monolayers were then scratched using a 10 µL-pipette tip. The medium was exchanged and the wound healing was registered by live video microscopy. Images were captured in 10×min intervals for 17 h using a ZEISS (light) microscope Axiovert 40C, linked to a CCD video camera (Hamamatsu). Image acquisition was controlled by HiPic 32 (High Performance Image Control System) or WASABI (Hamamatsu Imaging Software). The analysis of the wound healing was performed using the Java-based image processing program ImageJ. Assays were performed independently for three times.

### Analysis of the Cell Size

A549 cells expressing EPHB6-wild type, the EPHB6-mutant or low EPHB6 (control/empty vector transduced) were seeded into cell culture flasks which were precoated with a collagen matrix. The cells were allowed to adhere for five hours and then monitored using a ZEISS (light) microscope Axiovert 40C, linked to a CCD video camera (Hamamatsu). Image acquisition was controlled by HiPic 32 (High Performance Image Control System) or WASABI (Hamamatsu Imaging Software). The analysis of the cell size of single cells was performed as described previously [24], using the visualization software Amira and the Java-based image processing program ImageJ. The software analyze parameters of cellular functions from single cells, including the determination of the cell area (in µm^2^).

### Metastasis Experiments in vivo

For all mouse experiments, we used 8 to 10 weeks old NOD.CB17-Prkdc<scid>/J (NOD/SCID) mice obtained from Charles River. To analyze metastasis development upon intravenous tumor cell injection, 22 NOD/SCID mice were irradiated with a single dose of 3.5 Gy from a cobalt-60 unit 1 day before transplantation. A total of 3×10^5^ stably transfected cells (dissolved in 200 µl PBS) were injected intravenously into the tail vein. Four weeks after transplantation, the mice were sacrificed and the development of lung metastasis was analyzed. In two cases, mice died within one week after transplantation: one mouse transplanted with EPHB6-wt expressing A549 cells and one mouse transplanted with EPHB6-mutant expressing A549 cells. Metastasis development was evaluated by counting individual metastatic nodules. For histological analyses, the lungs were fixed in 4% paraformaldehyde. In all experiments, treatment groups were randomized to prevent cage effects.

### Statistical Analysis

All experimental data are shown as mean plus standard deviation if not indicated otherwise. The mean values of three groups were compared by OneWay ANOVA from Raw data or in case of two groups by the students t-test. A two-sided p<0.05 was considered significant.

## Results

### EPHB6 Variants in NSCLC

All exons of the entire coding region of EPHB6 were sequenced by Sanger based sequencing in a cohort of NSCLC patients. Among 80 NSCLC patients, three (3.8%) cases were found to have non-synonymous mutations of EPHB6 (R52C, n = 1; Q498H, n = 1; and DPG915-917del, n = 1). No EPHB6 mutations were identified in the cell lines A549, HTB56 or HTB58. A bioinformatics analysis by SIFT and Polyphen indicated the point mutations R52C and Q498H as probably damaging (Table 1). A database search of recent large scale sequencing efforts revealed additional mutations that so far have not been further investigated (Fig. 1A).

EPHB6 mutations were identified in various exons and functional domains of the gene. Intriguingly, the deletion del915-917 (Fig. 1B) was located adjacent to two mutations (D915G and G914V) recently identified in patients with colorectal cancer [25], [26]. The cluster of mutations in this region between the tyrosine kinase catalytic (Tyrkc) domain and sterile alpha motif (SAM) further suggests functional relevance. This mutation was identified in a patient with lung adenocarcinoma. The mutation was heterozygous and was also detected in corresponding normal lung tissue indicating a germline mutation. The other mutations were solely detected in the tumor and not in matched healthy tissue inficating a somatic mutation.

For functional assays, EPHB6-wildtype and several mutants (R52C, Q498H, del915-917 and the P728S mutation previously described in ovarian cancer [LIT]) were stably co-transfected with an EGFP-expressing plasmids into the NSCLC-cell line A549 that expresses very low levels of EPHB6. EGFP positive clones were picked and expression analysis was performed by western blotting using EPHB6 antibody on single clones and on bulk cultures (Fig. 2A). Despite successful transfection with increased mRNA levels, protein expression of most of the EPHB6 mutants did not increase beyond the levels observed in empty vector transfected cells (Fig. 1C). Only for the del915-917 mutation, protein expression at levels similar to EPHB6 wildtype was achieved (Fig. 2A). Due to the insufficiency of adequate protein expression of the R52C and G498H mutants and the previously described P728S mutant, we functionally focused on the del915-917 mutation in NSCLC in subsequent experiments.

### Effects of EPHB6 Mutations on Migration and Metastasis of NSCLC Cells

As reported, EPHB6 wildtype inhibited migration of stably transfected A549 cells in a transwell chamber (Boyden chamber) assay. In contrast, cells transfected with the del915-917 mutant EPHB6 migrated significantly faster in this assay (Fig. 2B). In addition, we performed scratch assays with repeated video microscopy to follow the scratch closure over time. Wildtype EPHB6 inhibited *in vitro* wound healing compared to the empty vector, whereas the EPHB6 mutant cells closed the gap much faster than the wildtype (three independent experiments, p<0.002, ANOVA; p<0.0004, t-test) (Fig. 2C, D). It appeared that the EPHB6 mutant did not just inhibit the EPHB6 wildtype activity, but accelerated the closing of the wound compared to empty vector and wildtype. After eight hours of adherence, the open area of the EPHB6 mutant cells starts to decrease two times (3%) faster as it could be recognized for the EPHB6 wildtype cells (1.6%). Even the control cells showed less acceleration of wound closure (2%) at this time point.

To analyze the *in vivo* effects of EPHB6 mutations on the metastatic capacity of NSCLC cells, we performed *in vivo* metastasis assays. NOD/SCID mice were intravenously injected with EPHB6-wt cells (n = 10), EPHB6-del915-917 cells (n = 10) and empty vector control cells (n = 2). Four weeks after transplantation mice were sacrificed and analyzed. One mouse of each group of mice transplanted with EPHB6-wt and EPHB6-mut expressing cells died within one week after transplantation. Mice injected with EPHB6 wild type overexpressing cells showed lower numbers of lung metastases compared to mice injected with either empty vector control cells or EPHB6-mutant cells (Fig. 3): one mouse was found without metastasis, 2 mice with each 1 or 3 metastasis and one mouse with each 4, 5 or 8 metastasis. One mouse transplanted with EPHB6 wild type cells was found with a high number of lung metastasis. Interestingly, in all mice injected with EPHB6 mutant cells lung metastasis were detectable (Fig. 3; p = 0.011, t-test of data from mice transplanted with EPHB6-wt compared to EPHB6-mut cells). An *in vitro* proliferation assay after 72 hours (Fig. 4A) showed that EPHB6 mutant cells did not differ from EPHB6 wildtype expressing cells in terms of proliferative activity. Similar results were obtained in proliferation assays analyzed after 48 hours (data not shown). The experiments rather suggested that the increased metastatic activity *in vivo* was associated with the alteration of intrinsic migratory properties. EPHB6 wildtype receptor expression did not significantly change the shape of cells (although the variation of shape size increased) whereas the size of EPHB6 mutant cells that grew on regular plastic dishes was significantly diminished (Fig. 4B; p<0.05, t-test of data from 20 cells of EPHB6-wt and EPHB6-mut expressing cells). In line with these findings, the chemotaxis of EPHB6 cells on plastic dishes appeared to be reduced, most likely due to reduced adhesion properties. But the differences were statistically not significant (data not shown).

## Discussion

Ephrin – Eph receptor interactions are frequently deregulated in cancer (Reference). In current study we identified mutations of EPHB6 as a pro-metastatic feature in non-small cell lung cancer. One mutation, del915-917, was also present in matched normal tissue, strongly suggesting a germline alteration. Germline alterations have previously been described for EPHB6 in familial colorectal cancer To date, the functional consequences of these genetic alterations on a cellular level are unknown [25].

Alterations of Eph receptors frequently occur in lung cancer. One large scale sequencing study found mutations in 10 out of 13 Eph receptor genes in lung adenocarcinoma [27]. Due to the multiplicity of Eph receptor associated signaling events and the complex networking of receptors, the functional outcome of Eph receptor aberrations remain unclear [28]. For most of the Eph receptor alterations identified to date, functional consequences have not been studied.

Several somatic mutations of the EPHB6 gene have been previously identified in lung cancer [27], colorectal cancer [25], [26], ovarian cancer [29] and glioma [26].

In this study, screening of 80 NSCLC patient samples and 3 NSCLC cell lines identified 3 previously unknown mutations for the EPHB6 gene. One of this mutations, del915-917, resides in the domain between the tyrosine kinase and the sterile alpha motif (SAM) domain, where 2 somatic mutations were recently identified in colorectal cancer [25], [26]. The function of this domain is suggested to be related to cancer, and our findings in this work do support this suggestion. The *in vivo* experiments show clearly that expression of the mutated EPHB6 enhanced metastasis. In addition EPHB6-mutant expressing cells showed a threefold enhanced transwell migration towards a serum gradient (chemotaxis). These results are consistent with our *in vivo* results. Mice transplanted with EPHB6-mut cells developed significantly (p = 0.011) more lung metastases as mice transplanted with EPHB6-wt cells. In addition to the altered functions of the EPHB6 del(915-917) mutant, a few aspects might also suggest a gain of function. For example, the patterns of wound healing differed between EPHB6 wildytpe and mutant. It is possible that signaling differences exist between the wildtype and the mutant receptor. On the other hand, it might also be interesting to speculate that the mutant receptor might act dominant negative towards other inhibitory EPH receptors. This dominant negative activity might lead to the observation of potential gain of function potency. Clearly, future studies might reveal the underlying differences in signaling and the influence of other member of the EPH and EPH-receptor networks. Future studies might also reveal the functional effects of the non-del915-917 mutations. It is likely that these also inactivate EPHB6 but this needs to be confirmed in the future.

Recently, we could demonstrate that EPHB6 is frequently silenced by epigenetic mechanisms in lung cancer [21], and others could show the same inactivation mechanism in breast cancer [14]. Our studies also indicated that loss of EPHB6 induces a highly metastatic phenotype. In line, EPHB6 is the receptor tyrosine kinase for which low expression was most closely related with poor prognosis in early stage non-small cell lung cancer [20]. EPHB6 might play an important role in lung cancer metastasis given that it is frequently epigenetically silenced and/or mutated in a significant fraction of patients. This makes it possible that EPHB6 is a relevant modifier of metastatic capacity in lung cancer.

Taken together, mutations in EPHB6 occurring in non-small cell lung cancer might lead towards a pro-metastatic phenotype. Loss of EPHB6 function by decreased expression or mutational inactivation might therefore contribute to lung cancer metastasis.
